# Factors Other than the Glomerular Filtration Rate That Determine the Serum Beta-2-Microglobulin Level

**DOI:** 10.1371/journal.pone.0072073

**Published:** 2013-08-22

**Authors:** Zeno Stanga, Stefan Nock, Pedro Medina-Escobar, Urs E. Nydegger, Martin Risch, Lorenz Risch

**Affiliations:** 1 Division of Endocrinology, Diabetes and Clinical Nutrition and Department of General Internal Medicine, University Hospital and University of Bern, Bern, Switzerland; 2 Private University, Triesen, Liechtenstein; 3 Labormedizinische zentren Dr. Risch, Liebefeld, Switzerland; 4 Zentrallabor, Kantonsspital, Chur, Switzerland; 5 Division of Clinical Biochemistry, Medical University Innsbruck, Innsbruck, Austria; Mario Negri Institute for Pharmacological Research and Azienda Ospedaliera Ospedali Riuniti di Bergamo, Italy

## Abstract

**Background:**

β2-microglobulin has been increasingly investigated as a diagnostic marker of kidney function and a prognostic marker of adverse outcomes. To date, non-renal determinants of β2-microglobulin levels have not been well described**.** Non-renal determinants are important for the interpretation and appraisal of the diagnostic and prognostic value of any endogenous kidney function marker.

**Methods:**

This cross-sectional analysis was performed within the framework of the www.seniorlabor.ch study, which includes subjectively healthy individuals aged ≥60 years. Factors known or suspected to have a non-renal association with kidney function markers were investigated for a non-renal association with serum β2-microglobulin. As a marker of kidney function, the Berlin Initiative Study equation 2 for the estimation of the estimated glomerular filtration rate (eGFR_BIS2_) in the elderly was employed.

**Results:**

A total of 1302 participants (714 females and 588 males) were enrolled in the study. The use of a multivariate regression model adjusting for age, gender and kidney function (eGFR_BIS2_) revealed age, male gender, and C-reactive protein level to be positively associated with β2-microglobulin levels. In addition, there was an inverse non-renal relationship between systolic blood pressure, total cholesterol and current smoking status. No association with markers of diabetes mellitus, body stature, nutritional risk, thyroid function or calcium and phosphate levels was observed.

**Conclusions:**

Serum β2-microglobulin levels in elderly subjects are related to several non-renal factors. These non-renal factors are not congruent to those known from other markers (i.e. cystatin C and creatinine) and remind of classical cardiovascular risk factors.

## Introduction

The glomerular filtration rate (GFR) is commonly regarded as the most reliable global index of kidney function [1]. There are two types of GFR values that differ based on the method used for assessment: the measured GFR (mGFR) and the estimated GFR (eGFR). The mGFR is commonly regarded as the most accurate value for assessing kidney function [2]. The mGFR is determined using exogenously administered substances, such as inulin, iohexol and different isotopes. However, for practical reasons, this parameter is rarely used in routine clinical practice [3]. To approximate the mGFR, kidney function can be estimated using kidney function equations, which are based on demographic data and standardized measurements of serum analytes, such as creatinine and cystatin C [4]–[7]. Concomitant reporting of the eGFR together with the serum analyte concentrations has become standard in many clinical laboratories [8].

Serum markers of kidney function have a reciprocal relationship with the GFR, i.e., serum marker levels increase with decreasing GFR and vice versa [2]. However, kidney function is not the only determinant of a marker’s serum concentration. Because of practical reasons, equations estimating eGFR account for the most important but not all non-renal factors. Therefore, even if the estimating equations for eGFR account for non-renal factors of kidney function, other non-renal markers can still exert an influence on the eGFR estimate.

Serum creatinine is influenced by age, gender, skin color and ethnicity, body habitus, chronic illness, nutritional status and diet [2], [9], [10]. The concentration of cystatin C is influenced by high-dose glucocorticoid therapy, thyroid function and, to a lesser degree, C-reactive protein (CRP), diabetes mellitus, systolic blood pressure, age, smoking, gender and serum concentrations of other analytes [11]–[16]. β2-microglobulin is a low-molecular-weight protein (11.81 kDa) that is a component of the MHC 1 molecule present on all nucleated cells [17]. Because of the dependency of its serum concentration on the GFR, β2-microglobulin is one of the three classical low-molecular-weight protein markers of kidney function, i.e., cystatin C, β-trace protein and β2-microglobulin [18]–[22]. Inflammatory conditions, glucocorticoid therapy and lymphoproliferative disease have been reported to be non-renal determinants of the serum β2-microglobulin concentration; the latter condition is also a rationale for the marker’s use as a tumor marker in lymphoproliferative disease [23]–[27]. Currently, little is known about other non-renal determinants. This issue merits further study because β2-microglobulin is attracting increasing interest as an endogenous serum marker of the GFR [28]–[30].

Because non-renal determinants are important for the interpretation and appraisal of the diagnostic and prognostic value of any endogenous kidney function marker, we aimed to study the association of non-renal factors with the serum β2-microglobulin concentration. Factors investigated for a non-renal association with kidney function markers in other studies were chosen for the present analysis.

## Subjects and Methods

### Study Population

The study participants were recruited from August 2009 to November 2010 within the context of the Seniorlabor Study, which is an ongoing investigation in the canton of Berne (Switzerland) aimed at establishing appropriate reference intervals of several analytes in the elderly (http://www.seniorlabor.ch). Consecutive subjectively healthy elderly volunteers aged 60 years and older were recruited, as previously described [31]. In brief, the study participants were contacted through newspaper advertisements, various clubs and associations that had high proportions of healthy elderly members (e.g., alpine clubs and sports clubs) and personal contacts of the collaborators of the study organization. Participants with previously known diabetes mellitus, a diagnosis of active neoplastic disease within the last 5 years, hospitalization during the preceding 4 weeks, previously known thyroid disease and those using glucocorticoid medications were not allowed to participate in the study. This study was conducted in accordance with the Declaration of Helsinki and approved by the canton’s ethical committee board (Kantonale Ethikkommission Bern). All of the participants provided written informed consent.

### Data Collection

Each participant’s personal history was collected, anthropometric measurements, such as height and weight, were taken, and blood pressure was measured in a sitting position after a 10-minute rest period. Venous blood was drawn into S-Monovettes® (Sarstedt, Sevelen, Switzerland) after an overnight fasting period. The laboratory samples were processed (i.e., centrifuged, aliquoted and analyzed or frozen at −80°C) immediately after the blood was drawn. The geriatric nutritional risk index (GNRI) score, modified by Yamada et al. [32], was used as an index of nutritional status [33], [34]. The GNRI is calculated as follows:
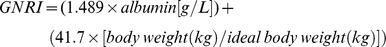



In this equation, the ratio of the actual body weight to the ideal body weight is set to one when the patient’s body weight exceeds the ideal body weight. Ideal body weight was defined as the value calculated from height and a BMI of 22 [32].

### Laboratory Methods

The laboratory parameters were determined with various analytical platforms. The β2-microglobulin concentration was determined using an Immulite 2000 (Siemens, Zurich, Switzerland).

The IDMS-standardized creatinine (alkaline picrate method) concentrations and albumin levels were determined on a Cobas Integra 800 instrument (Roche Diagnostics, Rotkreuz, Switzerland). The cystatin C level was determined using a nephelometric method on a Siemens ProSpec (Siemens, Zurich, Switzerland). The results were standardized to the International Federation of Clinical Chemistry and Laboratory Medicine standard, as described by Inker et al. [5], and the estimated GFR was determined using the Berlin Initiative Study equation 2 (BIS2), which is the only equation that has been evaluated for estimating GFR in elderly Caucasian subjects [7]. This equation accounts for age, gender and standardized cystatin C and creatinine results; in the elderly, it has been shown to outperform the original CKD-EPI equation for estimating GFR [5], [35]. However, this equation has only undergone internal validation in the original study and so far lacks external validation. The eGFR_BIS2_ is calculated as follows [7]:
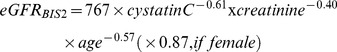



The level of high-sensitivity CRP was determined on a Siemens ProSpec (Siemens, Zurich, Switzerland). The concentrations of albumin, calcium, glucose, phosphate and potassium were assayed on a Cobas Integra 800 instrument (Roche Diagnostics, Rotkreuz, Switzerland). The hemoglobin and white blood cell (WBC) count were measured on a Sysmex XE-5000 hematology analyzer (Sysmex, Horgen, Switzerland). The thyroid-stimulating hormone (TSH) level was determined using an Architect 4000i (Abbott Diagnostics, Baar, Switzerland), and the hemoglobin A1c (HbA1c) level was determined using high-performance liquid chromatography (BioRad D10, Pratteln, Switzerland). The inter-day coefficients of variation, determined with commercially available control materials, were as follows: 7.30% at 1.22 mg/L and 8.32% at 3.18 mg/L for β2-microglobulin; 3.75% at 0.37 mg/L and 3.43% at 0.44 mg/L for cystatin C; 4.27% at 42 µmol/L and 1.96% at 556 µmol/L for creatinine; 3.7% at 24 g/L and 2.1% at 42 g/L for albumin; 6.7% at 0.64 mmol/L and 2.4% at 2.21 mmol/L for phosphate; 2.0% at 2.53 mmol/L and 2.0% at 3.25 mmol/L for calcium; 2.2% at 3.5 mmol/L and 1.9% at 6.5 mmol/L for glucose; 5.38% at 5.8 g/L and 4.87% at 44.7 g/L for CRP; 2.95% at 0.78 nU/L and 3.29% at 27.7 mU/L for TSH; and 2.88% at 5.7% and 2.38% at 10.8% for HbA1c.

### Statistical Analysis

The data are presented as medians and interquartile ranges (IQRs) or mean ± standard deviation, depending on the assessment with the Pearson d’Agostino test. To determine whether the β2-microglobulin concentrations were associated with non-renal factors, linear regression models were used. As candidate predictors, variables known to be independently from kidney function being associated with other endogenous markers of GFR [12], were investigated: such as age, gender, BMI, height, weight, systolic and diastolic blood pressure, self-reported hypertension, known cardiovascular disease, known musculoskeletal problems, GNRI, current smoking, blood urea nitrogen, hemoglobin, white blood cell count, glucose, hemoglobin A1_c_, potassium, sodium, total cholesterol, triglycerides, calcium, phosphate, albumin, CRP, and TSH. Continuous variables were log transformed if they were not normally distributed. These models considered β2-microglobulin as a dependent variable and other clinical and laboratory parameters as independent variables. Unadjusted associations were calculated. Furthermore, these associations were adjusted for kidney function, as assessed with eGFR_BIS2_, age and gender. Finally, a model employing all of the significant predictors in the aforementioned adjusted associations was fit. For the multivariate models, partial correlation coefficients r_partial_ were calculated. These coefficients estimated the correlation of each variable with log β2-microglobulin, adjusted for the effect of the other variables in the model. The comparisons between the two groups were performed with the Mann-Whitney U-test. Proportions were compared using the chi-square test. For all comparisons, *P* values <0.05 were considered to be significant. p-values up to 0.20 are reported numerically, whereas p-values >0.2 are reported as not significant (n.s.). All calculations were performed with MedCalc software version 12.2.1 (MedCalc software, Mariakerke, Belgium).

## Results

**Figure 1:**
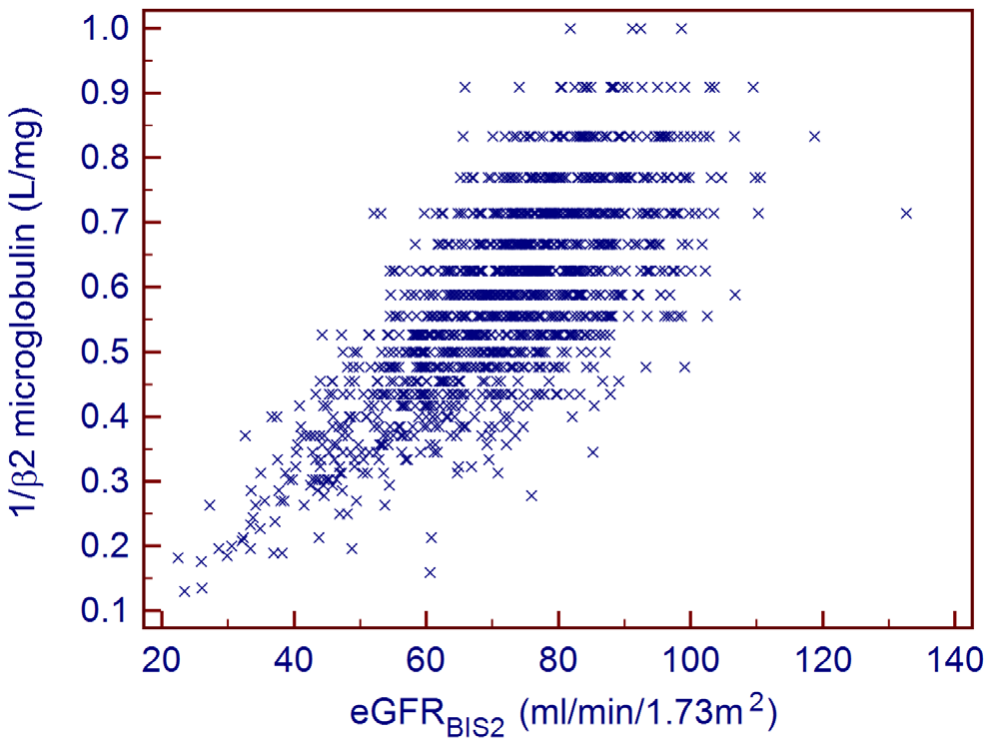
The relationship between β2-microglobulin and eGFR_BIS2_.

A total of 1302 individuals (714 female/588 male) with a median age of 72 years and an IQR of (66,78) were included in the study. All participants were of Caucasian descent. The detailed characteristics of the study population are given in Table 1. The median eGFR_BIS2_ was 73 ml/min/1.73 m^2^ (62,83). An eGFR_BIS2_<60 ml/min/1.73 m^2^ was present in 20.20% of the participants, whereas an eGFR_BIS2_>90 ml/min/1.73 m^2^ was found in 9.83%. There were no significant gender-specific differences in age, systolic and diastolic blood pressure, eGFR_BIS2_, albumin, HbA1_c_, potassium or CRP. However, female participants had a significantly lower BMI, weight, height, GNRI score, smoking prevalence, cystatin C level, creatinine level, β2-microglobulin level, blood urea nitrogen level, hemoglobin level, fasting glucose level and WBC count. Males had lower calcium and phosphate levels than females. Although statistically significant, these gender-specific differences, with the exception of serum creatinine and hemoglobin levels, were small. The Spearman rank correlation coefficient of the reciprocal of β2-microglobulin and eGFR_BIS2_ was 0.70 (*P*<0.001). The relationship between the inverse β2-microglobulin levels and eGFR_BIS2_ is given in Figure 1. Table 2 shows the regression coefficients together with standard errors relating the log β2-microglobulin with different predictor variables. As expected, the univariate analysis of β2-microglobulin levels and the chosen predictors hypothesized to be associated with kidney function markers revealed a significant relationship for a majority of predictors.

**Table 1:** Demographics of the study cohort stratified according to gender.

Variable	Female (*n = *714)Median (IQR)/N [%]	Male (*n = *588)Median (IQR)/N (%)	p
**Age (years)**	72 (66,79)	71 (66,78)	n.s.
**BMI (kg/m^2^)**	24.5 (22.1, 26.9)	25.4 (23.6,28.0)	<0.001
**Height (cm)**	163 (159,169)	174 (170,178)	<0.001
**Weight (kg)**	64 (58,72)	77 (71,86)	<0.001
**Systolic blood pressure (mmHg)**	146 (130,161)	148 (133,164)	0.09
**Diastolic blood pressure (mmHg)**	90 (80,100)	90 (80,99)	n.s.
**Self-reported Hypertension (N)**	279 (39.08)	256 (43.54)	0.12
**Cardiovascular disease (N)**	91 (12.75)	83 (14.12)	n.s.
**Musculosceletal disease (N)**	93 (13.03)	55 (9.35)	0.04
**HbA1c ≥6.5% (N)**	47 (6.58)	62 (10.54)	0.01
**GNRI score**	106 (103,108)	106 (103,109)	0.03
**Current smoker (N)**	37 (5.18)	63 (10.17)	<0.001
**eGFR_BIS2_ (ml/min/1.73 m^2^)**	72 (62,82)	73 (63,82)	0.11
**eGFR_CKD-EPI2012_ (ml/min/1.73 m^2^)**	81 (69. 92)	83 (71,93)	0.15
**β2-microglobulin (mg/L)**	1.7 (1.5,2.1)	1.8 (1.5,2.2)	0.05
**Cystatin C (mg/L)**	0.87 (0.78,1.00)	0.91 (0.82,1.03)	<0.001
**Creatinine (mg/dl)**	0.75 (0.67,0.85)	0.96 (0.86,1.07)	<0.001
**Blood urea nitrogen (mmol/L)**	5.2 (4.5,6.1)	5.9 (4.9,7.0)	<0.001
**Hemoglobin (g/L)**	137 (131,142)	149 (142,155)	<0.001
**Potassium (mmol/L)**	4.5 (4.2,4.7)	4.5 (4.3,4.7)	n.s.
**Glucose (mmol/L)**	5.1 (4.8,5.5)	5.4 (5.1,5.8)	<0.001
**Total cholesterol (mmol/L)**	6.1 (5.3,6.8)	5.4 (4.7,6.0)	<0.001
**Triglycerides (mmol/L)**	1.2 (0.9,1.6)	1.2 (0.9,1.7)	n.s.
**Calcium (mmol/L)**	2.40 (2.34,2.46)	2.37 (2.30,2.42)	<0.001
**Phosphate (mmol/L)**	1.18±0.14	1.03±0.14	<0.001
**Albumin (g/L)**	43 (42,45)	43 (42,45)	n.s.
**HbA1_c_ (%)**	5.8 (5.6,6.1)	5.8 (5.6,6.1)	n.s.
**CRP (mg/L)**	1.30 (0.68,2.50)	1.29 (0.71,2.33)	n.s.
**TSH (U/L)**	1.86 (1.25,2.57)	1.68 (1.22,2.44)	0.07
**WBC (cells/µl)**	5.3 (4.6,6.3)	5.7 (4.9,6.7)	<0.001

Values are shown as medians with IQRs in parentheses for continuous variables or numbers and percentages in brackets for categorical variables.

**Table 2:** Coefficients and standard errors from linear regression analysis assessing the relationship between log β2-microglobulin concentrations as dependent variable and several independent variables are shown.

	Unadjusted			Adjusted for log eGFR_BIS2_, log age and gender			
Variable	Coeff.	Std. error	p	Coeff.	Std. error	p	r_partial_
**Log age (years)**	1.175	0.065	<0.001	−0.254	0.061	<0.001	−0.11
**Male gender**	0.016	0.007	0.02	0.023	0.004	<0.001	0.14
**Log eGFR_BIS2_ (ml/min/1.73 m^2^)**	−0.969	0.023	<0.001	−1.054	0.030	<0.001	−0.70
**Log BMI (kg/m^2^)**	0.114	0.056	0.04	−0.048	0.037	0.19	–
**Log height (cm)**	−0.125	0.154	n.s.	−0.070	0.135	n.s.	–
**Log weight (kg)**	0.053	0.045	n.s.	−0.047	0.034	0.17	–
**Log systolic blood pressure (mmHg)**	−0.082	0.053	0.12	−0.091	0.035	0.01	−0.07
**Log diastolic blood pressure (mmHg)**	−0.212	0.055	0.001	−0.045	0.036	0.20	–
**Self-reported Hypertension**	0.061	0.007	<0.001	0.009	0.005	0.06	–
**Cardiovascular disease**	0.084	0.010	<0.001	0.009	0.007	0.16	–
**Musculosceletal disease**	0.021	0.011	0.06	−0.007	0.007	n.s.	–
**Log GNRI score**	−1.267	0.199	<0.001	−0.106	0.132	n.s.	–
**Current smoker**	−0.036	0.014	0.01	−0.020	0.009	0.03	−0.06
**Log blood urea nitrogen (mmol/L)**	0.466	0.026	<0.001	0.018	0.023	n.s.	–
**Log hemoglobin (g/L)**	−0.361	0.094	<0.001	−0.120	0.070	0.09	–
**Log potassium (mmol/L)**	0.266	0.097	0.006	−0.131	0.062	0.05	–
**Log glucose (mmol/L)**	0.148	0.056	0.008	−0.005	0.037	n.s.	–
**Log total cholesterol (mmol/L)**	−0.294	0.038	<0.001	−0.164	0.026	<0.001	−0.17
**Log triglycerides (mmol/L)**	0.085	0.018	<0.001	0.000	0.012	n.s.	–
**Log calcium (mmol/L)**	−0.035	0.195	n.s.	−0.187	0.126	0.14	–
**Phosphate (mmol/L)**	−0.031	0.021	0.15	−0.011	0.015	n.s.	–
**Log albumin (g/L)**	−0.868	0.125	<0.001	−0.035	0.084	n.s.	–
**Log HbA1_c_ (%)**	0.569	0.096	<0.001	0.036	0.064	n.s.	–
**Log CRP (mg/L)**	0.074	0.007	<0.001	0.031	0.005	<0.001	0.17
**Log TSH (U/L)**	0.010	0.012	n.s.	−0.001	0.008	n.s.	–
**Log WBC (cells/µl)**	0.217	0.033	<0.001	0.049	0.022	0.02	0.06

The coefficients for log age, male gender, and eGFR_BIS2_, the coefficients were obtained from a model incorporating log eGFR_BIS2_, log age and gender as independent variables and log β2-microglobulin as dependent variable. This model explained 60% of the variability of log β2-microglobulin. The partial correlation coefficients r_partial_ are given for significant predictors in the adjusted models and represent the correlation of each variable with log β2-microglobulin, adjusted for the effect of the other variables in the model (i.e. log eGFR_BIS2_, log age, and gender). *P* denotes the level of significance for each specific relationship.

Non-renal factors associated with log β2-microglobulin were assessed by adjusting the linear regression models for log age, gender and log eGFR_BIS2_. Fitting these models revealed that age, gender, systolic blood pressure, current smoking, potassium level, total cholesterol, CRP level and WBC count were non-renal predictors of β2-microglobulin concentrations. β2-microglobulin increased with age, male gender, CRP levels and WBC levels. In contrast, β2-microglobulin levels were inversely associated with increasing systolic blood pressure, total cholesterol and current smoking status. There did not seem to be a non-renal association between markers of diabetes mellitus, body stature, nutritional risk, thyroid function or calcium and phosphate levels and β2-microglobulin levels.

Finally a model incorporating all significant predictors from the aforementioned adjusted models was fitted, as shown in Table 3. With the exception of potassium and WBC levels, all predictors remained significant. The strongest association of β2-microglobulin concentrations, as obtained by partial correlation, was seen with kidney function. Weaker associations were seen with, age, gender, CRP, total cholesterol, systolic blood pressure, and smoking.

**Table 3:** A multivariate model including all the significant predictors of log β2-microglobulin concentrations from table 2 is shown.

Variable	Coeff	Std. error	p	r_partial_
**Log age (years)**	−0.241	0.064	<0.001	−0.11
**Male gender**	0.015	0.004	0.002	0.09
**Log eGFR_BIS2_ (ml/min/1.73 m^2^)**	−1.009	0.030	<0.001	−0.68
**Log systolic blood pressure (mmHg)**	−0.064	0.035	0.03	−0.06
**Current smoker**	−0.022	0.009	0.02	−0.07
**Log total cholesterol (mmol/L)**	−0.140	0.026	<0.001	−0.15
**Log CRP (mg/L)**	0.029	0.005	<0.001	0.16
**Log WBC (cells/µl)**	0.009	0.022	n.s.	–
**Log potassium (mmol/L)**	−0.080	0.061	n.s.	–

This model explains 62% of the variability of log β2-microglobulin concentrations and is thus not much more predictive than a model containing log eGFR_BIS2_, log age, and gender as independent variables. The partial correlation coefficients r_partial_ are given for significant predictors in the model and represent the correlation of each variable with log β2-microglobulin, adjusted for the effect of the other variables in the model.

## Discussion

This cross-sectional study in a population of subjectively health elderly participants predominantly having mild to moderate impairment of kidney function [1] provides evidence that the β2-microglobulin concentration, independent of kidney function, is associated with several factors, such as systolic blood pressure, age, gender, total cholesterol, markers of inflammation and smoking status. There were no relationships between β2-microglobulin and several non-renal factors known to be associated with other kidney function markers, such as cystatin C and creatinine. To our knowledge, this is the first study to investigate these associations in models adjusting for kidney function.

Understanding the non-renal determinants of an endogenous serum marker of kidney function is important because this understanding allows for the identification of variables to be used in the equations to transform a serum value into the corresponding eGFR [10]. The introduction of these variables into the equations improves both the precision and the accuracy of GFR estimates. Furthermore, information on the non-renal determinants of kidney function markers might also help to elucidate the relationship between the serum concentrations of these markers and clinical outcomes of interest, such as mortality and cardiovascular or renal incidents [10], [29], [36]. Accordingly, our study provides evidence that age and gender should be considered as predictors in regard to the evaluation of an equation for estimating GFR from β2-microglobulin [20]. This is analogous to the GFR estimation equations based on creatinine and/or cystatin C measurements [6], [37]. Adding the other significant parameters CRP, total cholesterol, systolic blood pressure, and smoking status into a kidney function equation, although increasing complexity of the equation, is not expected to substantially increase accuracy of kidney function estimates. However, in patients with current smoking, marked inflammation, marked hypertension, and marked hypercholesterolemia, a kidney function equation based on β2-microglobulin should be used with caution.

Interestingly, we did not find non-renal associations between β2-microglobulin and body weight or body mass index (BMI). This finding is in line with the findings described by Filler et al. [38], who investigated 216 pediatric urology patients aged 0.8–18 years. No changes in β2-microglobulin with age and weight were observed in these children. To our knowledge, however, this is the first investigation that reports the absent association between weight and β2-microglobulin in adults. We cannot offer an explanation for this absent relationship, which is in contrast to the findings of another low-molecular-weight protein, cystatin C, having been described to have a non-renal relationship with weight and BMI [12]. Creatinine, in contrast to cystatin C, has been shown to depend on dietary protein intake [10]. Our investigation used nutritional risk as a surrogate marker of protein intake and did not find a relationship between nutritional risk and β2-microglobulin levels. This is in line with the findings of Viberti et al. [39], who did not find a relationship between food intake and β2-microglobulin levels in 12 patients with diabetes mellitus.

Serum β2-microglobulin levels can increase as an acute phase reactant in several diseases. Accordingly, increased levels have been found in autoimmune diseases, such as systemic lupus erythematosus, rheumatoid arthritis and Sjögren’s syndrome, as well as in lymphoproliferative and infectious diseases [23], [25], [40]–[42]. The present investigation found that the association between inflammatory markers (CRP and WBC) and β2-microglobulin was independent of kidney function, even at normal levels. Inflammatory markers have also been reported to be associated with cystatin C but not with serum creatinine [12]. This additional association with inflammatory states could be one of the reasons why risk prediction for cardiovascular events and mortality is better with cystatin C and β2-microglobulin than with creatinine alone [29], [43], [44]. Further, when looking at the significant predictors of β2-microglobulin concentrations, it becomes apparent, that all these predictors also represent major cardiovascular risk factors (i.e. decreased kidney function, cholesterol, smoking, age, gender, increased high sensitive CRP, systolic blood pressure). Interestingly, not all these major cardiovascular risk factors exhibit an uniform positive non-renal association with β2-microglobulin concentrations. Some of them were also inversely correlated illustrating opposing effects of the different non-renal determinants of β2-microglobulin concentrations. Despite these opposing effects, however, other studies found β2-microglobulin concentrations to be significant non-renal predictors of cardiovascular outcomes, renal outcomes, and mortality [29], [36].

Our study identified smoking to be a non-renal factor associated with β2-microglobulin levels. Our multivariate model indicates that this association is independent from inflammation. This is in contrast to the report by Juraschek et al. [30], who did not find increased β2-microglobulin levels in smokers compared to non-smokers in the NHANES III survey. This finding, however, was obtained from logistic regression modeling, which did not adjust for kidney function markers. These somehow discrepant findings add to the controversy on current smoking as a non-renal factor associated with cystatin C. In the PREVEND cohort, Knight et al. [15] found a significant independent association between smoking and cystatin C, while White et al. [45] did not observe such a relationship in a cohort of kidney transplant patients. The non-renal association of systolic but not diastolic blood pressure with β2-microglobulin levels has not been described to our knowledge. This finding parallels the finding of the non-renal association of systolic blood pressure with cystatin C [12].

Non-renal determinants are important for the interpretation and appraisal of the diagnostic and prognostic value of any endogenous kidney function marker. For example, it does not make much sense to assess kidney function with a creatinine-based GFR estimate in patients with reduced muscle mass due to spinal cord injury. For this purpose, cystatin C has been shown to have better diagnostic characteristics than serum creatinine [46]. Conversely, it might be useless to assess kidney function using cystatin C-based GFR estimates in patients with thyroid disease and exposure to high doses of glucocorticoids [14], [47]. While our findings suggest that β2-microglobulin serves as an alternative protein marker to cystatin C in patients with thyroid disease, it would be useless in patients on glucocorticoids [26]. Table 4 summarizes different non-renal factors and their associations with serum levels of creatinine, cystatin C and β2-microglobulin. These results could lead to the patient-specific use of several kidney function markers. In a hypothetical patient with paraplegia and untreated thyroid disease, neither cystatin C nor creatinine would be expected to allow for unbiased kidney function assessment. β2-microglobulin could be the appropriate kidney marker for this patient.

**Table 4:** Non-renal factors associated with serum levels of endogenous kidney function markers.

Variable	Creatinine	Cystatin C	β2-Microglobulin
**Age**	+ [12]	+ [12]	+
**Gender**	+ [12]	+ [12]	+
**Ethnicity**	+ [12]	+ [12]	− [30]
**Diabetes/HbA1c/Glucose**	− [12]	+ [12]	−
**Systolic blood pressure**	− [12]	+ [12]	+
**Weight/BMI**	+ [12]	+ [12]	−
**Nutritional status/GNRI**	+ [10]	− [10]	−
**Lymphoproliferative disease**	− [52]	− [24]	+ [23]
**Current smoking**	?	+/− [15], [45]	+
**Glucocorticoid therapy**	− [45]	+ [14], [53]	+ [26]
**Calcineurin inhibitors**	− [45]	+/− [45], [54]	?
**Thyroid function**	− [55]	+ [47]	−
**Potassium**	− [12]	− [12]	+
**Hemoglobin**	− [12]	+ [12]	−
**Serum urea nitrogen**	+ [12]	− [12]	−
**WBC count**	+ [12]	+[12]	+
**CRP**	− [12]	+ [12]	+
**Phosphate**	+ [12]	− [12]	−
**Calcium**	− [12]	+ [12]	−
**Cholesterol**	− [12]	− [12]	+

Data were collected from the present study or the literature. References to other studies are given in brackets. Not all studies employed multivariate adjustments for evaluating non-renal associations. The method of assessing kidney function varied between the different studies.

However, more studies are necessary before the β2-microglobulin can be used as a kidney function marker in clinical routine. First, β2-microglobulin methods should be standardized [48], [49]. Second, a GFR estimating equation for standardized β2-microglobulin should be developed. To our knowledge, the equation by Donadio et al. [20] is the only GFR estimate based on β2-microglobulin that is currently available. This equation is based on a different assay for measuring the estimated GFR (Abbott Axsym) than the one employed in our study. Using the results from our method together with Donadio’s equation would lead to erroneous GFR estimates, as observed in Figure S1. As long as β2-microglobulin is not standardized, each assay should employ its corresponding GFR estimation equation [50].

Our study has several limitations. First and most importantly, we estimated GFR and did not use a reference method directly measuring GFR by means of exogenous substances. Direct GFR measurement does not undergo non-renal modification and allows for definitive answers. Logistically, it would have been impossible for us to perform direct GFR measurements in such a large population. Although we did not use a reference method for GFR assessment, we used the most accurate routine methods currently available, which rely on two serum markers and represent the appropriate methods for the population under study [7]. Furthermore, the first studies on non-renal factors associated with cystatin C levels did not employ reference methods for GFR assessment (e.g., glucocorticoid therapy, thyroid function, weight, CRP, gender and age) [14], [15], [47], [51]. These findings could be confirmed in studies employing reference methods for GFR assessment [12]. In this respect, we believe that our investigation adds important information on another endogenous kidney function marker. Second, the generalizability of our findings is hampered by the fact that we exclusively examined Caucasian subjects and that our participants were elderly. Our findings cannot be extrapolated to the general population. Nevertheless, it is in the elderly population, where the importance of kidney function estimation increases, in order to prevent errors in clinical management, especially regarding drug dosing or application of contrast media. Third, it may also be possible that we missed important non-renal factors. In summary, however, we believe that these limitations do not invalidate our findings.

## Conclusions

The serum concentration of β2-microglobulin was demonstrated to be associated with several non-renal factors, such as age, gender, CRP, cholesterol, systolic blood pressure, gender and current smoking. All these factors represent major cardiovascular risk factors. Our findings were derived from elderly individuals and cannot be extrapolated to the general population. Elderly patients, however, represent a collective with frequent need for determination of kidney function. Several factors affecting serum cystatin C levels, such as thyroid dysfunction, weight and hemoglobin concentrations, did not exhibit a non-renal association with β2-microglobulin. Information on non-renal factors associated with β2-microglobulin will allow for the identification of clinical scenarios where other kidney function markers, such as cystatin C and creatinine, are unable to provide adequate answers.

## Supporting Information

Figure S1 Introducing β2-microglobulin values from the Siemens Immulite into the equation by Donadio [20] leads to erroneous GFR estimates that systematically underestimate kidney function. A Bland-Altman plot is shown. This method assesses the agreement between two measurements of the same variable. Bias denotes the mean of all differences, whereas the limits of agreement are assessed with ±1.96 standard deviations around the bias. The mean bias is −10.1 ml/min/1.73 m^2^. The limits of agreement are −32.3 to +12.2 ml/min/1.73 m^2^. The eGFR_BIS2_ is used as a reference method for determining kidney function.
(TIF)
