# Supplementary figures and images for: Factors Other than the Glomerular Filtration Rate That Determine the Serum Beta-2-Microglobulin Level
Source: PLoS One. 2013 Aug 22;8(8):e72073. doi: 10.1371/journal.pone.0072073 (PMC3750024; doi:10.1371/journal.pone.0072073)

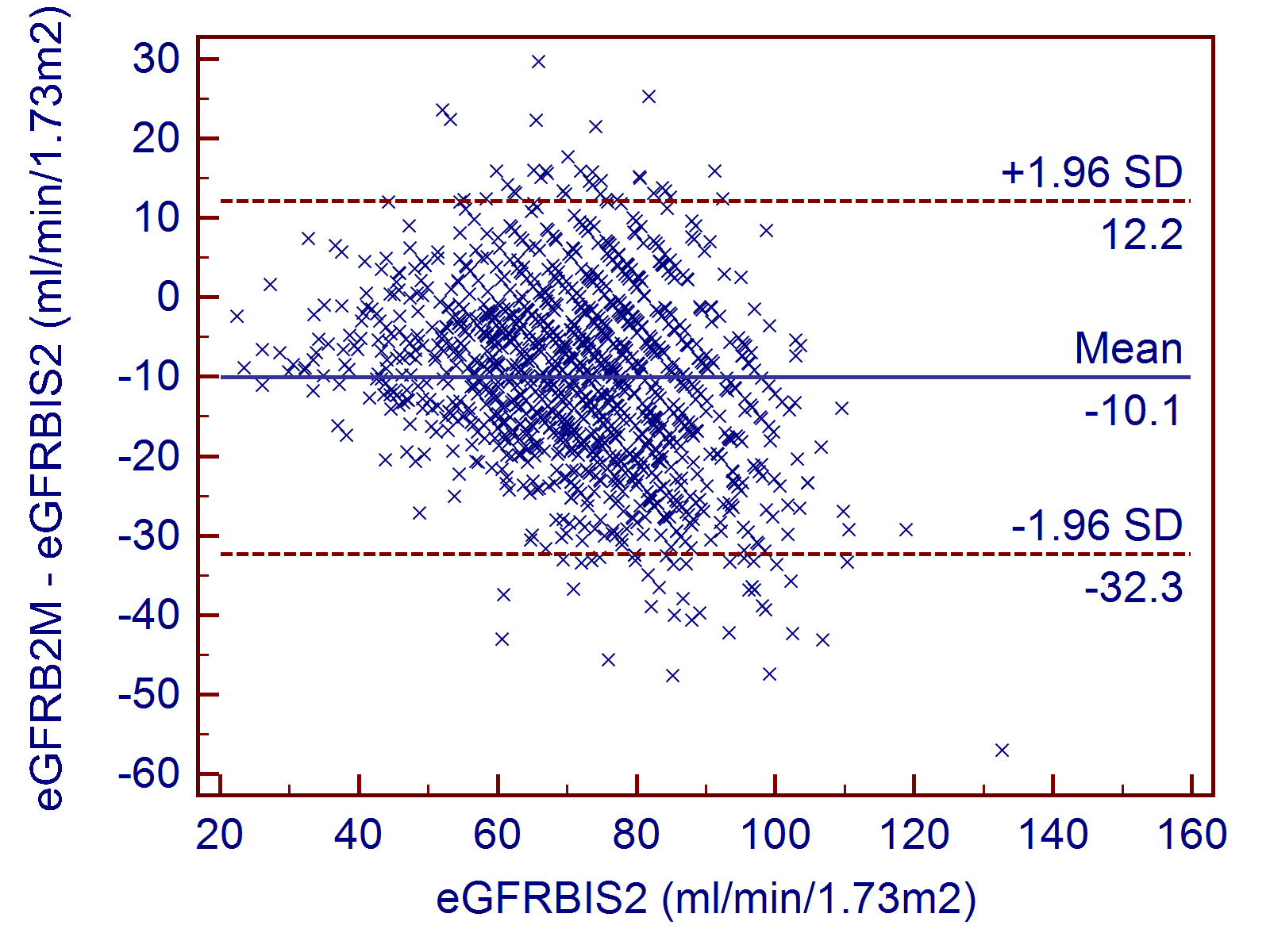

Supplement: Figure S1 — Introducing β2-microglobulin values from the Siemens Immulite into the equation by Donadio [20] leads to erroneous GFR estimates that systematically underestimate kidney function. A Bland-Altman plot is shown. This method assesses the agreement between two measurements of the same variable. Bias denotes the mean of all differences, whereas the limits of agreement are assessed with ±1.96 standard deviations around the bias. The mean bias is −10.1 ml/min/1.73 m2. The limits of agreement are −32.3 to +12.2 ml/min/1.73 m2. The eGFRBIS2 is used as a reference method for determining kidney function. (TIF) [file pone.0072073.s001.tif]
